# Analysis of Predictive Values Based on Individual Risk Factors in Multi-Modality Trials

**DOI:** 10.3390/diagnostics3010192

**Published:** 2013-03-15

**Authors:** Katharina Lange, Edgar Brunner

**Affiliations:** Department of Medical Statistics, University of Göttingen, Humboldtallee 32, 37073 Göttingen, Germany; E-Mail: ebrunne1@gwdg.de

**Keywords:** positive predictive value, negative predictive value, diagnostic trials, coronary artery disease

## Abstract

The accuracy of diagnostic tests with binary end-points is most frequently measured by sensitivity and specificity. However, from the clinical perspective, the main purpose of a diagnostic agent is to assess the probability of a patient actually being diseased and hence predictive values are more suitable here. As predictive values depend on the pre-test probability of disease, we provide a method to take risk factors influencing the patient’s prior probability of disease into account, when calculating predictive values. Furthermore, approaches to assess confidence intervals and a methodology to compare predictive values by statistical tests are presented. Hereby the methods can be used to analyze predictive values of factorial diagnostic trials, such as multi-modality, multi-reader-trials. We further performed a simulation study assessing length and coverage probability for different types of confidence intervals, and we present the R-Package *facROC* that can be used to analyze predictive values in factorial diagnostic trials in particular. The methods are applied to a study evaluating CT-angiography as a noninvasive alternative to coronary angiography for diagnosing coronary artery disease. Hereby the patients’ symptoms are considered as risk factors influencing the respective predictive values.

## 1. Introduction

The main purpose of a diagnostic agent is to assess a patient’s true health status, so the probability of the test giving the correct diagnosis is an important assessment of diagnostic ability. Hereby the *positive predictive value* describes the probability that a patient with an abnormal (*i.e*., positive) test result is actually diseased and consequently, the *negative predictive value* represents the probability that a patient with normal (*i.e*., negative) test result is actually free of disease. However, these quantities are only of limited value: the predictive values of a diagnostic agent critically depend on the prevalence of the disease and, as the prevalence might vary, e.g., between different risk groups, predictive values are not homogenous within the population. Hence *sensitivity* and *specificity* are mostly used to describe the accuracy of a diagnostic agent because these measures are independent of the prevalence of disease: they are defined as the probabilities of the test, correctly identifying the diseased subjects or the non-diseased respectively. In contrast to the predictive values, sensitivity and specificity describe the result of a test within groups of patients who either have or do not have the condition. Thus they are characteristics of the diagnostic test itself and are independent of the prevalence of the disease. Even though sensitivity and specificity are useful and powerful measures to understand how effective a diagnostic test is, they also involve a main disadvantage: these quantities do not assess the accuracy of a diagnostic agent in a practically useful way. They concentrate on how accurate the diagnostic test is in discriminating diseased and non-diseased subjects but fail to give an assessment of a normal or abnormal test result of an individual patient. This interpretation of the test result is only provided by predictive values. Hence, predictive values should not be disregarded in the analysis of diagnostic trials, despite all entailed problems. Instead of avoiding predictive values, they should rather be estimated while carefully taking the arising problems into consideration.

This paper now provides a new method to calculate predictive values for different risk groups. As the pre-test probability of disease in different risk groups does not need to be determined in the same study as the efficiency of the diagnostic agent, this method can even be applied after the diagnostic study (analyzed by means of sensitivity and specificity) has already been closed. Hence, the approach is also applicable to case-control studies where, per study design, no prevalence can be assessed. By this method of analysis, the heterogeneity of predictive values throughout the population is taken into account because predictive values are estimated for each risk group separately. Reported additionally to sensitivity and specificity, these predictive values provide a comprehensive review of the strengths and weaknesses of the investigated diagnostic agent.

In our approach of analysis, we assume that sensitivity and specificity are equal in all investigated risk groups and that the probability of disease in each group has been estimated in prior studies. We calculate predictive values by using Bayes’ theorem and determine the asymptotic distribution of the resulting inferential statistics by using the delta method (Section 2). As many diagnostic studies are at least two armed trials in which each subject is diagnosed by different tests, we use the multivariate delta theorem to allow to determine predictive values in factorial designs as well. The idea of using the delta method to calculate the distribution of predictive values in a univariate set-up was proposed by Mercaldo I. [1]. In their work the prevalence has to be known by the investigator and cannot be estimated, which seems to be unlikely in clinical practice. In our approach we hence allow the prevalence being a random variable, which cannot easily be neglected as simulations studies (Section 3) show. The practical relevance of presented methods are illustrated by means of a study evaluating the accuracy of multidetector CT angiography in the diagnosis of coronary artery disease (Section 4). The paper closes with a discussion of the proposed procedures (Section 6).

## 2. Methods of Analysis

In this approach, Bayes’ theorem [2] serves as the theoretical basis for the analysis. This theorem connects sensitivity and specificity with the predictive values by displaying the positive (*p*_+_) and the negative (*p_−_*) predictive value as functions of sensitivity (*se*), specificity (*sp*) and prevalence (*π*), namely,

(1) $$p+=f_{+}(se,sp,\pi )=\frac{se\cdot \pi }{se\cdot \pi +(1-sp)\cdot (1-\pi )}$$

(2) $$p_{-}=f_{-}(se,sp,\pi )=\frac{sp\cdot (1-\pi )}{sp\cdot (1-\pi )+(1-se)\cdot \pi }$$

The sensitivity can be estimated by the ratio of the true positive test results to all diseased subjects, and the specificity can be estimated by the ratio of the true negative test results to all non-diseased subjects. As we assume that the prevalence of disease in each risk group has been assessed in prior studies, estimators for the positive and the negative predictive value for each risk group can be calculated by plugging in the estimators of sensitivity, specificity and prevalence in Equations (1) and (2).

This method is applied, e.g., by Diamond and Forrester [3], who compute the probability of having a coronary artery disease. They present a table of post-test probabilities depending on the result of an electrocardiographic stress test (depression of the S-T segment) and depending on the pre-test probability of disease (categorized into different risk groups by age, sex and symptoms). Their work shows the importance of distinguishing between different risk groups: for the same depression of the S-T segment, the positive predictive value varies from 0.938 (high risk group) to 0.003 (low risk group).

Extending the ideas of Diamond and Forrester, our approach will go one step further: we will derive methods of analysis by which not only the predictive values of two or more modalities are calculated for different risk groups, but also the difference between the modalities is statistically tested. Furthermore, different methods to calculate confidence intervals for the positive and the negative predictive value are provided. As all these methods take the patient’s risk factors into consideration, this approach of analysis can be regarded as a further step towards personalized medicine.

### 2.1. Notation

We consider a diagnostic trial involving *N* subjects, where *n*_0_ subjects are classified as non-diseased by a reliable gold standard and *n*_1_ are classified as diseased. In the set-up of this trial, we assume that each subject is examined by means of *m* = 1, …, *M* different diagnostic tests. For each subject the results are collected in a vector $X_{ik}=\left(X_{ik}^{\left(1\right)},\ldots ,X_{ik}^{\left(M\right)}\right)$, *i* = 0, 1, *k* = 1, …, *n_i_*, where $X_{ik}^{\left(m\right)}=1$, if the test result of the *m*-th test is positive and $X_{ik}^{\left(m\right)}=0$ otherwise. Within group *i* (*i* = 0, 1) the vectors X*_ik_*, *k* = 1, …, *n_i_* are independent identically distributed random vectors, following a multivariate Bernoulli distribution with success probabilities $\mathrm{sp}=\left(sp^{\left(1\right)},\ldots ,sp^{\left(M\right)}\right)\prime$ for *i* = 0 and $\mathrm{se}=\left(se^{\left(1\right)},\ldots ,se^{\left(M\right)}\right)\prime$ for *i* = 1. Hereby sp and se denote the vectors of sensitivity and specificity for the different modalities.

### 2.2. Estimation and Asymptotic Distribution

The sensitivity of the *m*-th diagnostic test is estimated by ${\hat{se}}^{\left(m\right)}$, the ratio of the true positive test results (of test *m*) to all diseased subjects, and the specificity of the *m*-th modality is estimated by ${\hat{se}}^{\left(m\right)}$, the ratio of the true negative test results (of test *m*) to all non-diseased subjects. Similarly to sensitivity and specificity, their estimators are also collected in vectors $\hat{\mathrm{se}}={\left({\hat{se}}^{\left(1\right)},\ldots ,{\hat{se}}^{\left(M\right)}\right)}^{\prime }$ and $\hat{\mathrm{sp}}={\left({\hat{sp}}^{\left(1\right)},\ldots ,{\hat{sp}}^{\left(M\right)}\right)}^{\prime }$.

For the calculation of the predictive values for a subject, its pre-test probability of disease is required. We assume that the pre-test probability of disease is influenced by a patient’s individual characteristics and that each subject can be attributed to a risk group on the basis of these attributes. Hereby, the prevalences *π_g_, g* = 1, …, *G*, in the *g*-th risk group have been estimated in prior studies by ${\hat{\pi }}_{g}=\frac{k_{g}}{m_{g}}$, the ratio of the number of diseased subjects *k_g_* in group *g* to all subjects *m_g_* in group *g*. With the help of Equations (1) and (2), the positive and the negative predictive value of the *m*-th modality for b the *g*-th risk group can be calculated and finally be estimated by replacing sensitivity, specificity and prevalence by their respective estimates:

$$\begin{array}{l} {\hat{p}}_{g,+}^{\left(m\right)}=f+\left({\hat{se}}^{\left(m\right)},{\hat{sp}}^{\left(m\right)},{\hat{\pi }}_{g}\right)=\frac{{\hat{se}}^{\left(m\right)}\cdot {\hat{\pi }}_{g}}{\hat{se}\cdot {\hat{\pi }}_{g}+\left(1-{\hat{sp}}^{\left(M\right)}\right)\cdot \left(1-{\hat{\pi }}_{g}\right)}\;\text{and}\\ {\hat{p}}_{g,-}^{\left(m\right)}=f_{-}\left({\hat{se}}^{\left(m\right)},{\hat{sp}}^{\left(m\right)},{\hat{\pi }}_{g}\right)=\frac{{\hat{sp}}^{\left(m\right)}\cdot \left(1-{\hat{\pi }}_{g}\right)}{{\hat{sp}}^{\left(m\right)}\cdot \left(1-{\hat{\pi }}_{g}\right)+\left(1-{\hat{se}}^{\left(m\right)}\right)\cdot {\hat{\pi }}_{g}},\;m=1,\ldots ,M \end{array}$$

Similarly to sensitivity and specificity, the positive and the negative predictive values of each risk group *g* = 1, …, *G* are collected in vectors $p_{g,+}=\left(p_{g,+}^{\left(1\right)},\ldots ,p_{g,+}^{\left(M\right)}\right)$ and $p_{g,-}=\left(p_{g,-}^{\left(1\right)},\ldots ,p_{g,-}^{\left(M\right)}\right)$.

To derive the asymptotic results for the predictive values, the following regularity assumptions are required:

#### Assumptions

1. For all *l, r* = 1, …, *d* the bivariate distribution of $\left(X_{ik}^{\left(l\right)},X_{ik}^{\left(r\right)}\right)$ is the same for all subjects *k* = 1, …, *n_i_* within group *i*, *i* = 0, 1.
2. *Ñ* = min(*n*_0_, *n*_1_, *m_g_, g* = 1, …, *G*) *→ ∞*: Such that $\frac{N}{n_{i}}\to d_{i}$, *i* = 0, 1, and $\frac{N}{m_{g}}\to e_{g}$, *g* = 1, …, *G* as *N* → ∞.
3. *se*^(^*^l^*^)^, *sp*^(^*^l^*^)^ *l* = 1, …, *d* and *π_g_, g* = 1, …, *G* are in (0, 1).

In clinical practice, these assumptions can be interpreted in the following way. The first assumption means that different subjects are independent replications. The second assumption ensures that the sample sizes *n*_1_ (used for the estimation of sensitivity), *n*_0_ (used for the estimation of specificity) and *m_g_, g* = 1, …, *G* (used for the estimation of prevalences for the different risk groups) increase uniformly when the overall sample size is increased. The third assumption excludes the trivial case that the sensitivity, specificity or prevalences are equal to 0 or 1.

These assumptions lead to our main result:

#### Theorem

For each risk group *g* = 1, *… G*, the statistics $\sqrt{N}\left({\hat{p}}_{+}^{g}-p_{+}^{g}\right)$ and $\sqrt{N}\left({\hat{p}}_{-}^{g}-p_{-}^{g}\right)$*√* have, asymptotically, a multivariate normal distribution with mean 0 and covariance matrices ${\hat{V}}_{+}^{g}$ and ${\hat{V}}_{-}^{g}$, which are defined in Appendix A.

#### Proof

The proof is mainly based on the central limit theorem and Cramer’s multivariate delta theorem with *f*_+_ and *f_−_* as transformation functions. For details as well as for the expressions of ${\hat{V}}_{+}^{g}$ and ${\hat{V}}_{-}^{g}$ we refer the Appendix A.

The idea of using the delta method to calculate the distribution of predictive values was already proposed by Mercaldo *et al*. [1] for the univariate case, *i.e*., in their approach, predictive values of different diagnostic tests cannot be compared if the tests are carried out on the same subjects. It is further assumed that the prevalence is a known parameter but no quantity that has been estimated. If their method is applied in a set-up, when the prevalence is estimated (but incorrectly treated as fixed in order to meet the requirements for their approach), the variance of the predictive values is systematically underestimated (see Section 3).

### 2.3. Inferential Statistics

Based on the asymptotic distribution of $\sqrt{N}\left({\hat{p}}_{+}^{g}-p_{+}^{g}\right)$ and $\sqrt{N}\left({\hat{p}}_{-}^{g}-p_{-}^{g}\right)$, the usual test statistics to compare the different diagnostic tests can be statistically tested by formulating the hypotheses in the same way as in theory of linear models

$$H_{0}:p_{g,\pm }^{\left(m\right)}-{\bar{p}}_{g,\pm }=0,\forall m=1,\ldots ,M,\;\text{where}\;{\bar{p}}_{g,\pm }=\frac{1}{M}\sum_{l=1}^{M}p_{g,\pm }^{\left(l\right)}$$

which equivalently can be written as:

$$H_{0}:T\cdot p_{\pm }^{g}=0,\;\text{where}\;T=I_{M}-\frac{1}{M}1_{M}1_{M}^{\prime }$$

Hereby, I*_M_* denotes the *M*-dimensional unit matrix and 1*_M_* the *M*-dimensional vector of 1s. In this case, an additive model is assumed but this approach can easily be expanded to a logistic model by again applying the delta method with a logit-transformation function. Hypotheses can be tested with the help of the ANOVA-type statistic ([4,5]): Under *H*_0_ the statistic

$$ATS=\frac{N}{tr\left(T{\hat{V}}_{\pm }^{g}\right)}{\left({\hat{p}}_{\pm }^{g}\right)}^{\prime }T{\hat{p}}_{\pm }^{g}$$

can be approximated by a central $\chi_{\hat{f}}^{2}/\hat{f}$–distribution with

$$\hat{f}=\frac{{\left[tr\left(T{\hat{V}}_{\pm }^{g}\right)\right]}^{2}}{tr\left({\left[T{\hat{V}}_{\pm }^{g}\right]}^{2}\right)}$$

degrees of freedom. Furthermore, the (1 −* α*)-confidence intervals for each modality as well as for the difference between two modalities can be calculated in the usual way:

$$\begin{array}{l} \;{\hat{p}}_{g,\pm }^{\left(m\right)}\pm z_{1-\frac{\alpha }{2}}\frac{\sqrt{{\hat{v}}_{\pm }^{g}\left[m,m\right]}}{\sqrt{N}}\\ {\hat{p}}_{g,\pm }^{\left(m_{1}\right)}-{\hat{p}}_{g,\pm }^{\left(m_{2}\right)}\pm z_{1-\frac{\alpha }{2}}\frac{\sqrt{{\hat{v}}_{\pm }^{g}\left[m_{1},m_{1}\right]+{\hat{v}}_{\pm }^{g}\left[m_{2},m_{2}\right]-2{\hat{v}}_{\pm }^{g}\left[m_{1},m_{2}\right]}}{\sqrt{N}} \end{array}$$

where ${\hat{v}}_{\pm }^{g}\left[i,j\right]$ denotes the (*i, j*)-element of ${\hat{V}}_{\pm }^{g}$ and $z_{1-\frac{\alpha }{2}}$ the $\left(1-\frac{\alpha }{2}\right)$-quantile of a standard normal distribution. For the confidence intervals, as well as for the test statistic, a logistic model can be applied. Hereby, the logistic model has one main advantage: the resulting confidence intervals are range-preserving by construction.

For small sample sizes, the distribution of $\sqrt{N}\left({\hat{p}}_{g,\pm }^{\left(m\right)}-p_{g,\pm }^{\left(m\right)}\right)/\sqrt{{\hat{v}}_{\pm }^{g}\left[m,m\right]}$ can be approximated by a central *t_ν_*-distribution (see Appendix B), which increases the coverage probability of the resulting confidence intervals.

## 3. Simulation Results

In this section, we investigate the coverage and length of confidence intervals constructed with the delta method. Hereby, we compare the approach of Mercaldo *et al*. [1] with the approaches presented in this paper. There were 48 different combinations for prevalence *π* ∈ {0.05, 0.25, 0.5}, sensitivity and specificity *se, se* ∈ {0.5, 0.75, 0.85, 0.9}. Three different values of *n* = *n*_0_ = *n*_1_ ∈ {50, 100, 500} and *m_g_* ∈ {100, 500, 1, 000} were used with each combination to symbolize small, medium and large study sizes for both the study evaluating the usefulness of the diagnostic test and the study assessing the prevalence of disease according to risk groups. For each combination of *π, se, sp, n, m_g_*, 10,000 binomial samples were generated using the function rbinom of the free software **R** [6]. Hereby in each simulation step, the sensitivity was estimated from a contingency table generated by *n*_1_ Bernoulli samples, the specificity was estimated from a contingency table generated by *n*_0_ Bernoulli samples, and the prevalence was estimated by means of *m_g_* Bernoulli samples. The positive and negative predictive values as well as their estimators were calculated by applying Bayes’s theorem to a given set of *π, se, sp* and $\hat{\pi }$, $\hat{se}$, $\hat{sp}$, respectively.

Simulation results for *p*_+_ and *p_−_* can be found in Tables 1 and 2 and Tables A1 and A2, respectively. Hereby, the results for the negative predictive value are presented in Appendix C for reasons of readability. Due to the great number of input parameters and the number of possible values, these tables were constructed in the same way as in the paper of Mercaldo *et al*. [1]: one parameter was held fixed while averaging over the remaining parameters. If the positive or the negative predictive value is estimated 0 or 1, the logistic confidence interval is not applicable. In this case the *t_ν_*-approximation is not applicable neither, because the denominator of *ν* is estimated to be 0. The number of times this occurred was recorded in the last columns of Tables 1 and A1, respectively. As the point estimators for *p*_+_ and *p_−_* are equal in all approaches the failure rates of the t-approximation, the logistic normal approximation, the logistic t-approximation and the logistic method by Mercaldo *et al*. are the same.

The approach of Mercaldo *et al*. assumes that the prevalence is a known fixed parameter and the variance of *π_g_* is 0. But as the prevalence can only be assessed by estimation, we assumed the prevalence to be a binomial random variable with variance greater than 0. To investigate whether this assumption has an impact on the quality of the confidence intervals or whether this assumption can easily be neglected in practice, we also simulated the approach of Mercaldo *et al*. with *π_g_* being a random variable. Note that we hence investigate the methodology of Mercaldo *et al*. in set-up, which seems likely in clinical practice but for which it was not designed. As the assumption that *π_g_* is known by the investigator seems to be unlikely in practice, no simulation with fixed *π_g_* was performed.

Simulation results show that the logistic confidence intervals have a slightly higher coverage probability than the additive intervals. The t-approximation in the additive set-up seems to achieve the best coverage.

For our approach the overall coverages for the logistic interval are 0.9568 (*p*_+_) and 0.9562 (*p_−_*), whereas the overall coverages for the additive interval are 0.9364 (*p*_+_) and 0.9303 (*p_−_*). The t-approximation increases coverage such that the overall coverages achieve 0.9494 (*p*_+_) and 0.9440(*p_−_*). The t-approximated logistic confidence intervals tend to be even more conservative than the normal-approximated logistic confidence intervals. Furthermore, simulation results show that the assumption of π_g_ being a fixed parameter is a necessary assumption for a good performance of the approach of Mercaldo *et al*. If π_g_ is a random variable, the overall coverage probability only reaches 0.7885 (additive) or 0.7998 (logistic) for the positive predictive value. (Simulations of the negative predictive values lead to comparable results.) The variance of ${\hat{\pi }}_{g}$ decreases when the sample size *m_g_* increases and, hence, the method of Mercaldo *et al*. achieves better results for large *m_g_*.

As their approach assumed the variance of *π_g_* equal to 0, the lengths of the confidence intervals are noticeably smaller than in our approach (about 25%), whereas for both methods the additive and logistic approaches yield comparable intervals lengths. For each method, the lengths of the logistic and the additive confidence intervals are almost equal. By construction, the lengths of the t-approximated confidence intervals are slightly higher than the intervals constructed by means of the normal approximation, but the difference is negligible.

We hence recommend using either the t-approximation or the logistic normal approximation when confidence intervals are computed.

## 4. Applications: Diagnostic Performance of Multidetector CT Angiography

As coronary artery disease (CAD) has been recognized as the leading cause of death in the United States [7], the diagnosis of the presence and severity of CAD is essential in clinical practice. Conventional coronary angiography reveals the extent, location and severity of obstructive lesions with high accuracy and thus invasive coronary angiography, despite the associated risks, remains the standard procedure for the diagnosis of CAD. Multidetector computed tomographic angiography (MDCTA) has been proposed as a noninvasive alternative to the conventional coronary angiography.

Recently, Miller *et al*. [8] performed a multi-center diagnostic trial to evaluate the accuracy of MDCTA involving 64 detectors. In 291 patients, segments of 1.5 mm or more in diameter were analyzed by means of CT and conventional angiography (gold standard) to assess whether the patient has at least one coronary stenosis of 50% or more. These data are summarized Table 3.

From Table 3, the sensitivity is estimated to be 0.85 while the specificity is estimated to be 0.90. Miller *et al*. [8] also estimated the predictive values from Table 3. Hereby, they implicitly assumed that the pre-test likelihood of disease is the same for all patients. They, furthermore, assumed that the study prevalence is representative. Using this approach, 0.83 is estimated as positive predictive value and 0.91 as negative predictive value. From these results, the authors draw the conclusion that CT angiography cannot be used as a simple replacement for conventional angiography. For our approach of analysis, we will regard three risk groups with different pre-test probabilities of disease. Diamond and Forrester [3] reviewed the literature to estimate the prevalence of CAD depending on sex, age and symptoms. For reasons of simplicity, we will only concentrate on the patient’s symptoms as risk factor. According to the patient’s symptoms, Diamond and Forrester [3] provide the pre-test probabilities of disease presented in Table 4.

Using these estimators for the prevalence as well as the estimators of sensitivity and specificity, the positive predictive values (PPV), negative predictive values (NPV) and the corresponding confidence intervals were calculated using the described methods. The results are summarized in Table 5.

Taking the additional information of the patient’s individual risk factors of disease into account offers a more comprehensive interpretation of the study results. For a patient with nonanginal chest pain, a negative test result from the MDCTA eliminates the need of further examination as well as a positive test result for a patient with typical angina does. In contrast, for a patient with atypical angina, neither a positive nor a negative test result from the MDCTA will lead to a clear statement concerning the patient’s health status.

## 5. Software

In order to analyze factorial trials, we have developed the R-Package facROC. The software can be used to evaluate most assessments of diagnostic accuracy in factorial set-ups: the area under the ROC-Curve (according to [9]), sensitivity and specificity (according to [10]) as well as predictive values (according to this paper). In most diagnostic trials sensitivity and specificity are analyzed as primary assessments of diagnostic accuracy. The evaluation of sensitivity and specificity serves as a basis for the computation of predictive values and can be performed by the facROC function facBinary:

fB <- facBinary(formula, id, gold, data, logit=FALSE)

Hereby the factorial structure of the trial can be taken into consideration with the help of the formula parameter that specifies the model in the usual way (e.g., formula = testresult ^~^ rater∗method). The parameter “id” indicates the patient’s id and the parameter “gold” assigns the patient’s true health status. (For more details as well as more options and parameters, see facROC manual.) To calculate and evaluate predictive values, the result of the analysis of sensitivity and specificity (*i.e*., a facBinary object) can be passed to the facPV function:

facPV(fB, prev, logit=FALSE, test=FALSE)

The prevalence parameter “prev” has to be passed to the facPV function as a two-dimensional vector: prev = c(diseased patients in prevalence study, number of patients). The options “logit” and “test” are logical flags indicating whether a logistic model should be fitted and whether hypotheses on the predictive values should be tested.

If the data of the original study determining sensitivity and specificity is not available and hence the function facBinary cannot be called, the function facPV can be used instead:

facPV(se, sp, n1, n0, prev, logit=FALSE)

Hereby “se” denotes a vector of sensitivities under different conditions and “sp” denotes the corresponding vector of specificities. Note that it is also possible to pass one-dimensional vectors to the function facPV. “n1” and “n0” characterize the sample sizes of diseased and non-diseased patients used to estimate “se” and “sp”. Again the logical flag “logit” indicates whether logistic confidence intervals should be computed. If the parameters to determine predictive values are provided without a facBinary object, the test option is not available. As the covariance matrixes of sensitivity and specificity are not at hand in this case, the test statistic cannot be computed: it summarizes predictive values of different conditions and these might be dependent if the corresponding sensitivities and specificities are dependent. As confidence intervals are computed for each condition separately, they can nevertheless be calculated.

The package facROC will shortly be available on CRAN. Currently it is uploaded at http://github.com/KatharinaLange. To install directly from github, the package devtools is needed (available on CRAN).

install.packages("devtools")

library(devtools)

install_github(repo="facROC", username="KatharinaLange")

The build Linux (tar.gz) and Windows (zip) versions of this package are available for download in the repository at http://github.com/KatharinaLange/facROC-build.

## 6. Discussion

In this paper, we suggested a new method to translate the results of diagnostic trials for use in clinical practice by means of predictive values. The proposed method provides an approach to calculate confidence intervals according to factors influencing the risk of disease. As in our approach the pre-test probability of disease has been assessed in prior studies, no prevalence needs to be estimated from the data of the current trial. Thus this method of analysis can also be used for calculating predictive values in case-control studies where estimating prevalences is not possible. Note that in our approach, we assume that the prevalence is independent of sensitivity and specificity, which means that sensitivity and specificity have to be homogeneous in different risk groups. Thus, with this methodology, it is possible to estimate predictive values for risk groups that are not included in the original trial. Note that the assumption of homogeneity has to be considered carefully before this method is applied (For example, the accuracy of imaging devices as well as the risk of disease might depend on the patient’s BMI. In this case, sensitivity and specificity are no longer equal in the different risk groups and hence a stratified estimation for each group has to be performed). We, furthermore, considered a set-up in which it is possible to compare the predictive values of different diagnostic tests by means of the ANOVA-Type statistic. Many diagnostic trials are imaging studies and therefore the investigation of the images is mostly carried out by several readers. As our approach uses the multivariate delta theorem, this method of analysis can easily be extended to multiple reader diagnostic trials by using a vector of indices (*r, m*) indicating the reader and the method. Hypotheses can be tested by choosing appropriate contrast matrices referring to the theory of linear models. Furthermore, confidence intervals for arbitrary contrasts $c\prime p_{\pm }^{g}$ can be computed. Hence, in multiple reader trials we can assess the difference between two diagnostic tests by averaging over the different readers [9,10]. In clinical practice, an initial suspicion is sometimes confirmed not by only one diagnostic test but by several ones. In this case, the pre-test probability of disease increases with each positive diagnostic result. In order to calculate and analyze predictive values in these cases, some information is required:

1. the probability of disease before the first test was carried out, *i.e*., the “*pre-testing*” probability of disease, which might depend on several risk factors and has to be determined from prevalence studies;
2. the sensitivity and the specificity of each diagnostic test performed as well as the correlation between these tests.

With the help of the second item, a global sensitivity and a global specificity for the whole testing procedure can be calculated. (This might be a complex problem if different diagnostic tests are dependent. For more details, see, e.g., [11].) In combination with the pre-testing probability of disease, predictive values can now be calculated in the way proposed here. Note that a pre-test or a pre-testing probability is always required when predictive values are computed. The methodology developed might help to answer two of the most important questions to clinicians: *“How likely is it that the patient has the condition?”* and *“How likely is it that the patient is free of disease?”* Nevertheless, it is important to point out that predictive values cannot replace sensitivity and specificity. As predictive values have a more concrete and thus more user-friendly interpretation than sensitivity and specificity, they might also be considered as accuracy assessments, when the usefulness of a new diagnostic agent is evaluated. However, because these measurements depend on the prevalence, regulatory authorities advise to be careful when using predictive values for the evaluation of diagnostic trials. The EMEA states *“predictive values must be reported with caution and only when the study sample is considered to be representative of the prevalence in the real world”* [12] and the FDA recommends that *“the trials include the intended population in the appropriate clinical setting”* [13]. Following these recommendations, predictive values are calculated for a patient with a mean pre-test risk of disease but the results of the evaluation will not be valid for a patient with a known higher or lower probability of disease. Hence, we achieve a result for an average patient but no general result. But the main purpose of a diagnostic trial is to evaluate whether or not a new diagnostic agent increases the probability of a correct diagnosis in general. In contrast, sensitivity and specificity are able to assess the effect of a new diagnostic agent independent of any prior probability of disease and any prevalence. Thus sensitivity and specificity allow us to assess the quality of a new diagnostic agent in general. Therefore, predictive values should rather be avoided when the usefulness of a new diagnostic agent is evaluated and they should only be calculated for the use in clinical practice.

## Figures and Tables

**Table 1:** Summary of *p*_+_ coverage probabilities where the cell values denote the coverage probability for one fixed parameter and averaging over the remaining parameters. Hereby N-Approx and t-Approx are abbreviations for normal and t-approximation. *π* fix denotes the method of [1], where *π* is assumed to be fixed.

Fixed Parameter	N-Approx	Additive t-Approx	*π* fix	N-Approx	Logistic t-Approx	*π* fix	Failure (logistic& t-Approx)
*π* = 0.05	0.9298	0.9525	0.6958	0.9608	0.9657	0.7013	0.234 %
*π* = 0.25	0.9403	0.9482	0.8250	0.9548	0.958	0.8386	0.045%
*π* = 0.5	0.9392	0.9472	0.8449	0.9548	0.9576	0.8596	0.047%
*se* = 0.5	0.9360	0.9477	0.8067	0.9571	0.9606	0.8184	0.105%
*se* = 0.75	0.9365	0.9496	0.7875	0.9569	0.9605	0.7985	0.111%
*se* = 0.85	0.9366	0.9498	0.7810	0.9568	0.9603	0.7925	0.107%
*se* = 0.9	0.9366	0.9501	0.7790	0.9564	0.9602	0.7900	0.111%
*sp* = 0.5	0.9408	0.9500	0.6897	0.9544	0.9557	0.6909	0.060%
*sp* = 0.75	0.9394	0.9480	0.7856	0.9552	0.9577	0.7913	0.061%
*sp* = 0.85	0.9357	0.9473	0.8287	0.9574	0.9623	0.8427	0.077%
*sp* = 0.9	0.9299	0.9520	0.8501	0.9602	0.9658	0.8745	0.235%
*m_g_* = 100	0.9266	0.9512	0.6578	0.9605	0.9646	0.6698	0.233%
*m_g_* = 500	0.9404	0.9476	0.8314	0.9552	0.9587	0.8425	0.046%
*m_g_* = 1, 000	0.9422	0.9491	0.8764	0.9547	0.9579	0.8873	0.047%
*n*_0_ = *n*_1_ = 50	0.9307	0.9498	0.8693	0.9608	0.9656	0.8915	0.197%
*n*_0_ = *n*_1_ = 100	0.9373	0.9474	0.8317	0.9563	0.9608	0.8416	0.065%
*n*_0_ = *n*_1_ = 500	0.9413	0.9508	0.6646	0.9533	0.9548	0.6664	0.063%
Overall	0.9364	0.9494	0.7885	0.9568	0.9603	0.7998	0.1080%

**Table 2:** Summary of *p*_+_ confidence interval lengths where the cell values denote the confidence interval length for one fixed parameter and averaging over the remaining parameters. Hereby N-Approx and t-Approx are abbreviations for normal and t-approximation. *π* fix denotes the method of [1], where *π* is assumed to be fixed.

Fixed Parameter	N-Approx	Additive t-Approx	*π* fix	N-Approx	Logistic t-Approx	*π* fix
*π* = 0.05	0.2045	0.2204	0.1290	0.2041	0.2216	0.1269
*π* = 0.25	0.2262	0.2326	0.1782	0.2224	0.229	0.1761
*π* = 0.5	0.1522	0.1554	0.1219	0.1533	0.1578	0.1232
*se* = 0.5	0.2006	0.2084	0.1561	0.1994	0.2081	0.1545
*se* = 0.75	0.1941	0.2027	0.1423	0.1932	0.2028	0.1414
*se* = 0.85	0.1918	0.2006	0.1379	0.1908	0.2007	0.1371
*se* = 0.9	0.1907	0.1995	0.1358	0.1896	0.1996	0.1351
*sp* = 0.5	0.1323	0.1343	0.0772	0.1329	0.1368	0.0770
*sp* = 0.75	0.1816	0.1856	0.1285	0.1815	0.1873	0.1277
*sp* = 0.85	0.2181	0.2270	0.1680	0.2165	0.2264	0.1666
*sp* = 0.9	0.2452	0.2642	0.1984	0.2421	0.2607	0.1968
*m_g_* = 100	0.2480	0.2610	0.1415	0.2476	0.2644	0.1407
*m_g_* = 500	0.1741	0.1802	0.1437	0.1727	0.1785	0.1426
*m_g_* = 1, 000	0.1607	0.1671	0.1439	0.1595	0.1654	0.1428
*n*_0_ = *n*_1_ = 50	0.251	0.2664	0.2142	0.2484	0.2641	0.212
*n*_0_ = *n*_1_ = 100	0.1959	0.2017	0.1492	0.1951	0.2021	0.1484
*n*_0_= *n*_1_ = 500	0.136	0.1402	0.0658	0.1363	0.1421	0.0657
Overall	0.1943	0.2028	0.1430	0.1933	0.2028	0.1420%

**Table 3:** CAD data.

		Conventional positive	Angiography negative
MDCTA	positive	140	13
	negative	24	114
		164	127

**Table 4:** Prevalence of CAD in symptomatic patients.

Symptom	Proportion of Patients Affected
nonanginal chest pain	146/913 (16.0%)
atypical angina	963/1,931 (49.9%)
typical angina	1,874/2,108 (88.9%)

**Table 5:** Predictive values of CAD according to symptoms.

	Nonanginal Chest Pain		Atypical Angina		Typical Angina	

	PPV 0.614	NPV 0.97	PPV 0.892	NPV 0.86	PPV 0.985	NPV 0.434
standard	[0.483,0.744]	[0.958,0.982]	[0.842,0.943]	[0.814,0.907]	[0.977,0.993]	[0.336,0.532]
with t-approx.	[0.480,0.747]	[0.957,0.982]	[0.840,0.945]	[0.814,0.907]	[0.977,0.993]	[0.335,0.533]
logistic	[0.478,0.733]	[0.955,0.980]	[0.830,0.933]	[0.807,0.901]	[0.975,0.991]	[0.339,0.533]
with t-approx	[0.474,0.736]	[0.955,0.980]	[0.828,0.935]	[0.807,0.901]	[0.975,0.991]	[0.338,0.534]

## A. Proof: Asymptotic Distribution of *p*_+_ and *p_−_*

According to the multivariate central limit theorem, we obtain that

(3) $$\sqrt{n_{1}}\cdot (\hat{\mathrm{se}}-\mathrm{se})\overset{ℒ}{\to }\tilde{U}\sim N\left(0,V_{se}\right)$$

where V*_se_* denotes the covariance matrix of X_11_. Applying the central limit theorem to the estimator of the specificities similarly leads to

(4) $$\sqrt{n_{0}}\cdot (\hat{\mathrm{sp}}-\mathrm{sp})\overset{ℒ}{\to }W\sim N\left(0,V_{sp}\right)$$

where V*_sp_* = *Cov*(X_01_). For the prevalences in the different risk groups, the univariate central limit theorem leads to

(5) $$\sqrt{m_{g}}\cdot \left({\hat{\pi }}_{g}-\pi_{g}\right)\overset{ℒ}{\to }Q_{g}\sim N\left(0,\sigma_{g}^{2}\right),g=1,\ldots ,G$$

where $\sigma_{g}^{2}=\pi_{g}\left(1-\pi_{g}\right)$. As $\frac{N}{n_{i}}\to d_{i}$, *i*=0, 1 and $\frac{N}{m_{g}}\to e_{g}$, *g* = 1, …, *G* by assumption, Equations (3)–(5) can be rewritten as

$$\begin{array}{l} \sqrt{N}\cdot (\hat{\mathrm{se}}-\mathrm{se})\overset{ℒ}{\to }U\prime \prime \sim N\left(0,d_{1}\cdot V_{se}\right)\\ \sqrt{N}\cdot (\hat{\mathrm{sp}}-\mathrm{sp})\overset{ℒ}{\to }W\prime \prime \sim N\left(0,d_{0}\cdot V_{sp}\right),\;\text{and}\\ \sqrt{N}\cdot \left({\hat{\pi }}_{g}-\pi_{g}\right)\overset{ℒ}{\to }Q\prime \prime \sim N\left(0,e_{g}\cdot \sigma_{g}^{2}\right),g=1,\ldots ,G \end{array}$$

The estimators of sensitivity, specificity and prevalence are independent random variables and thus we obtain that:

$$\sqrt{N}\left[\left(\begin{array}{c} \hat{\mathrm{se}}\\ \hat{\mathrm{sp}}\\ \hat{\pi } \end{array}\right)-\left(\begin{array}{c} \mathrm{se}\\ \mathrm{sp}\\ \pi_{g} \end{array}\right)\right]\overset{ℒ}{\to }B\sim N\left(0,d_{1}V_{se}⊕d_{2}V_{sp}⊕e_{g}\sigma_{g}^{2}\right)$$

where ⊕ denotes the direct sum.

The functions *f*_+_ and *f_−_* given in Equations (1) and (2) map sensitivity, specificity and the prevalence onto the positive and the negative predictive value, respectively. Let

$$\begin{array}{l} f_{+}\left(\left(\mathrm{se}\prime ,\mathrm{sp}\prime ,\pi_{g}\right)\prime \right)=\left(f_{+}\left(se^{\left(1\right)},sp^{\left(1\right)},\pi_{g}\right),\ldots ,f_{+}\left(se^{\left(d\right)},sp^{\left(d\right)},\pi_{g}\right)\right)\;\text{and}\\ f_{-}\left(\left(\mathrm{se}\prime ,\mathrm{sp}\prime ,\pi_{g}\right)\prime \right)=\left(f_{-}\left(se^{\left(1\right)},sp^{\left(1\right)},\pi_{g}\right),\ldots ,f_{-}\left(se^{\left(d\right)},sp^{\left(d\right)},\pi_{g}\right)\right) \end{array}$$

denote the multivariate versions of *f*_+_ and *f_−_* and let **Df**+ = **Df**+ ((se′, sp′, *π_g_*)′) and **Df***_−_* = **Df***_−_* ((se′, sp′, *π_g_*)′) denote the corresponding Jacobian matrices of all first-order partial derivatives at position (se′, sp′, *π_g_*)′. Then, applying Cramer’s *δ* theorem leads to:

$$\begin{array}{r} \sqrt{N}\left({\hat{p}}_{+}^{g}-p_{+}^{g}\right)=\sqrt{N}\left[f_{+}\left(\begin{array}{c} \hat{\mathrm{se}}\\ \hat{\mathrm{sp}}\\ {\hat{\pi }}_{g} \end{array}\right)-f_{+}\left(\begin{array}{c} \mathrm{se}\\ \mathrm{sp}\\ \pi_{g} \end{array}\right)\right]\overset{ℒ}{\to }B_{+}^{g}\sim N\left(0,Df_{+}\left[d_{1}V_{se}⊕d_{0}V_{sp}⊕e_{g}\sigma_{g}^{2}\right]Df_{+}^{\prime }\right)\\ g=1,\ldots ,G \end{array}$$

and

$$\begin{array}{r} \sqrt{N}\left({\hat{p}}_{-}^{g}-p_{-}^{g}\right)=\sqrt{N}\left[f_{-}\left(\begin{array}{c} \hat{\mathrm{se}}\\ \hat{\mathrm{sp}}\\ {\hat{\pi }}_{g} \end{array}\right)-f_{-}\left(\begin{array}{c} \mathrm{se}\\ \mathrm{sp}\\ \pi_{g} \end{array}\right)\right]\overset{ℒ}{\to }B_{-}^{g}\sim N\left(0,Df_{-}\left[d_{1}V_{se}⊕d_{0}V_{sp}⊕e_{g}\sigma_{g}^{2}\right]Df_{-}^{\prime }\right)\\ g=1,\ldots ,G \end{array}$$

Now **Df**+ = **Df**+ ((**se**′, **sp**′, *π_g_*)′) and **Df**− = **Df**− ((**se′, sp′**, *π_g_*)′) are estimated by ${\hat{\mathrm{Df}}}_{+}=Df_{+}\left(\left(\hat{\mathrm{se}}\prime ,\hat{\mathrm{sp}}\prime ,{\hat{\pi }}_{g}\right)\prime \right)$ and ${\hat{\mathrm{Df}}}_{-}=Df_{-}\left(\left(\hat{\mathrm{se}}\prime ,\hat{\mathrm{sp}}\prime ,{\hat{\pi }}_{g}\right)\prime \right)$, respectively. The quantities *d_i_* are estimated by $\frac{N}{n_{1}}$, *i* = 0, 1 and *e_g_* estimated by $\frac{N}{m_{g}}$, all *g=1,…, G.* We further estimate the covariance matrices **V***_se_* and **V***_sp_* by the sample covariance matrices

$${\hat{V}}_{se}=\frac{1}{n_{1}-1}\sum_{k=1}^{n_{1}}\left(X_{1k}-\hat{\mathrm{se}}\right)\left(X_{1k}-\hat{\mathrm{se}}\right)\prime$$

and

$${\hat{V}}_{sp}=\frac{1}{n_{0}-1}\sum_{k=1}^{n_{0}}\left(X_{0k}-\hat{\mathrm{sp}}\right)\left(X_{0k}-\hat{\mathrm{sp}}\right)\prime$$

respectively. We further use the unbiased empirical variance ${\hat{\sigma }}_{g}^{2}=\frac{m_{g}}{m_{g}-1}\cdot {\hat{\pi }}_{g}\cdot \left(1-{\hat{\pi }}_{g}\right)$ as the estimator of $\sigma_{g}^{2}$ for the ease of convenience.

Plugging in these empirical counterparts and applying Slutzky’s theorem hence leads to our main result: for each risk group *g* = 1, *… G*, the statistics $\sqrt{N}\left({\hat{p}}_{+}^{g}-p_{+}^{g}\right)$ and $\sqrt{N}\left({\hat{p}}_{-}^{g}-p_{-}^{g}\right)$ have, asymptotically, a multivariate normal distribution with mean 0 and covariance matrices

$$\begin{array}{l} {\hat{V}}_{+}^{g}={\hat{\mathrm{Df}}}_{+}\left[\frac{N}{n_{1}}{\hat{V}}_{se}⊕\frac{N}{n_{0}}{\hat{V}}_{sp}⊕\frac{N}{m_{g}}{\hat{\sigma }}_{g}^{2}\right]{\hat{\mathrm{Df}}}_{+}^{\prime }\;\text{and}\\ {\hat{V}}_{-}^{g}={\hat{\mathrm{Df}}}_{-}\left[\frac{N}{n_{1}}{\hat{V}}_{se}⊕\frac{N}{n_{0}}{\hat{V}}_{sp}⊕\frac{N}{m_{g}}{\hat{\sigma }}_{g}^{2}\right]{\hat{\mathrm{Df}}}_{-}^{\prime } \end{array}$$

respectively.

## B. t-Approximation for Small Sample Sizes

In order to increase the quality of our methods for small sample sizes, a *t_ν_*-approximation of $\sqrt{N}({\hat{p}}_{g,\pm }^{\left(m\right)}-p_{g,\pm }^{\left(m\right)})/\sqrt{{\hat{u}}_{\pm }^{g}[m,m]}$ is also provided. To assess the degrees of freedom *ν* of the t-distribution, we use an approach based on the Box approximation [14]: the distribution of $\sqrt{{\hat{v}}_{\pm }^{g}[m,m]}$ is approximated by a scaled $\chi_{\nu }^{2}$-distribution, *i.e*., by the distribution of a random variable *g · Z_ν_*, where $Z_{\nu }\sim \chi_{\nu }^{2}$ and *ν* and *g* are constants such that the first two moments coincide. We hence determine *ν* by

(6) $$\begin{array}{l} \nu =\frac{2\cdot E{\left(\frac{\partial f}{\partial se}\cdot d_{1}\cdot {\hat{v}}_{se}[m,m]+\frac{\partial f}{\partial sp}\cdot d_{0}\cdot {\hat{v}}_{sp}[m,m]+\frac{\partial f}{\partial \pi }\cdot e_{g}\cdot {\hat{\sigma }}_{g}^{2}\right)}^{2}}{\mathrm{Var}\left(\frac{\partial f}{\partial se}\cdot d_{1}\cdot {\hat{v}}_{se}[m,m]+\frac{\partial f}{\partial sp}\cdot d_{0}\cdot {\hat{v}}_{sp}[m,m]+\frac{\partial f}{\partial \pi }\cdot e_{g}\cdot {\hat{\sigma }}_{g}^{2}\right)}\\ \;=\frac{2\cdot {\left(\frac{\partial f}{\partial se}\cdot d_{1}\cdot E\left({\hat{v}}_{se}[m,m]\right)+\frac{\partial f}{\partial se}\cdot d_{0}\cdot E\left({\hat{v}}_{sp}[m,m]\right)+\frac{\partial f}{\partial \pi }\cdot e_{g}\cdot E\left({\hat{\sigma }}_{g}^{2}\right)\right)}^{2}}{\frac{\partial f}{\partial se}\cdot d_{1}\cdot \mathrm{Var}({\hat{v}}_{se}[m,m])+\frac{\partial f}{\partial se}\cdot d_{0}\cdot \mathrm{Var}({\hat{v}}_{sp}[m,m])+\frac{\partial f}{\partial \pi }\cdot e_{g}\cdot \mathrm{Var}({\hat{\sigma }}_{g}^{2})} \end{array}$$

where ${\hat{v}}_{se}[m,m]$ and ${\hat{v}}_{se}[m,m]$ denote the empirical variances of the $X_{ik}^{\left(m\right)},k=1,\ldots ,n_{i}$ for *i* = 1 and *i* = 0. ∂*f*/∂*se* is the partial derivative of *f* with respect to *se* at (*se^(m)^, sp^(m)^, π_g_*)′. The partial derivatives ∂*f*/∂*sp* and ∂*f*/∂*π* are defined analogously. As *ν* contains unknown parameters, *ν* itself is unknown and it has to be estimated. We, hence, estimate the partial derivatives at (*se^(m)^, sp^(m)^, π_g_*)′ by the partial derivatives at (*se^(m)^, sp^(m)^, π_g_*)′ and further estimate *d_i_* by *N/n_i_*, *i* = 0, 1 and *e_g_* by *N/e_g_*. As ${\hat{v}}_{se}[m,m]$, ${\hat{v}}_{sp}[m,m]$ and ${\hat{\sigma }}_{g}^{2}$ are unbiased by construction, the numerator can be determined easily by means of these plug-in estimates. For the denominator, we can estimate the variance of each term separately because the three parts of the sums are independent. We hence have to estimate the variances of

$$\begin{array}{c} {\hat{v}}_{se}[m,m]=\frac{1}{n_{1}-1}{\sum_{k=1}^{n_{1}}\left(X_{1k}^{\left(m\right)}-{\bar{X}}_{1\cdot }^{\left(m\right)}\right)}^{2}\\ {\hat{v}}_{sp}[m,m]=\frac{1}{n_{0}-1}{\sum_{k=1}^{n_{0}}\left(X_{0k}^{\left(m\right)}-{\bar{X}}_{0\cdot }^{\left(m\right)}\right)}^{2}\;\text{and}\\ {\hat{\sigma }}_{g}^{2}=\frac{m_{g}}{m_{g}-1}\cdot {\hat{\pi }}_{g}\cdot (1-{\hat{\pi }}_{g}) \end{array}$$

Note that ${\hat{\sigma }}_{g}^{2}$ also is the empirical variance of a Bernoulli distributed random variable and therefore can be represented in the same manner as ${\hat{v}}_{se}[m,m]$ and ${\hat{v}}_{sp}[m,m]$. Hence, we only have to determine the variance of the empirical variance of a Bernoulli distributed random variable, which is illustrated by the example of ${\hat{v}}_{se}[m,m]$. By defining the bin (*n*_1_, *se*)-distributed random variable $S_{1}=\sum_{k=1}^{n_{1}}X_{1k}^{\left(m\right)}$, ${\hat{v}}_{se}[m,m]$ can be represented as

$$\begin{array}{l} {\hat{v}}_{se}[m,m]=\frac{1}{n_{1}-1}\sum_{k=1}^{n_{1}}{\left(X_{1k}^{\left(m\right)}-{\bar{X}}_{1\cdot }^{\left(m\right)}\right)}^{2}\\ \;=\frac{1}{n_{1}-1}\left[\sum_{k=1}^{n_{1}}X_{1k}^{\left(m\right)}+\frac{1}{n_{1}}{\left(\sum_{k=1}^{n_{1}}X_{1k}^{\left(m\right)}\right)}^{2}\right]=\frac{1}{n_{1}-1}\left[S_{1}+\frac{1}{n_{1}}S_{1}^{2}\right] \end{array}$$

We, therefore, obtain for the variance of ${\hat{v}}_{se}[m,m]$:

$$\begin{array}{l} \mathrm{Var}({\hat{v}}_{se}[m,m])=\frac{1}{{\left(n_{1}-1\right)}^{2}}\left[E\left({\left[S_{1}-\frac{1}{n_{1}}S_{1}^{2}\right]}^{2}\right)-{\left(E\left[S_{1}-\frac{1}{n_{1}}S_{1}^{2}\right]\right)}^{2}\right]\\ \;=\frac{1}{{\left(n_{1}-1\right)}^{2}}\left[E(S_{1}^{2})-\frac{2}{n_{1}}E(S_{1}^{3})+\frac{1}{n_{1}^{2}}E(S_{1}^{4})-E(S_{1}^{4})-E{\left(S_{1}\right)}^{2}+\frac{2}{n_{1}}E(S_{1})\cdot E(S_{1}^{2})-\frac{1}{n_{1}^{2}}E{\left(S_{1}^{2}\right)}^{2}\right] \end{array}$$

As the first four moments of a binomial distribution can easily be determined, the estimator of *ν* can be obtained by plugging in all estimates in Equation (6).

## C. Simulation Results for the Negative Predictive Value

**Table A1:** Summary of *p*_−_ coverage probabilities where the cell values denote the coverage probability for one fixed parameter and averaging over the remaining parameters. Hereby N-Approx and t-Approx are abbreviations for normal and t-approximation. *π* fix denotes the method of [1], where *π* is assumed to be fixed.

Fixed Parameter	N-Approx	Additive t-Approx	*π* fix	N-Approx	Logistic t-Approx	*π* fix	Failure (logistic & t-Approx)
*π* = 0.05	0.9389	0.9471	0.8441	0.9546	0.9576	0.8590	0.047%
*π* = 0.25	0.9357	0.9437	0.8231	0.9548	0.9580	0.8384	0.040%
*π* = 0.5	0.9163	0.9411	0.6851	0.9592	0.9638	0.6982	0.244%
*se* = 0.5	0.9393	0.9484	0.6872	0.9540	0.9554	0.6900	0.064%
*se* = 0.75	0.9346	0.9432	0.7816	0.9547	0.9573	0.7901	0.064%
*se* = 0.85	0.9278	0.9399	0.8235	0.9567	0.9617	0.8408	0.076%
*se* = 0.9	0.9195	0.9446	0.8441	0.9593	0.9648	0.8733	0.245%
*sp* = 0.5	0.9307	0.9434	0.8031	0.9564	0.9598	0.8173	0.114%
*sp* = 0.75	0.9301	0.9439	0.7828	0.9562	0.9598	0.7974	0.114%
*sp* = 0.85	0.9304	0.9445	0.7769	0.9562	0.9598	0.7913	0.110%
*sp* = 0.9	0.9300	0.9442	0.7736	0.9559	0.9596	0.7881	0.112%
*m_g_* = 100	0.9178	0.9416	0.6536	0.9595	0.9636	0.6665	0.244%
*m_g_* = 500	0.9351	0.9435	0.8267	0.9544	0.9578	0.8421	0.047%
*m_g_* = 1, 000	0.9380	0.9468	0.8720	0.9547	0.9580	0.8870	0.046%
*n*_0_ = *n*_1_ = 50	0.9217	0.9433	0.8613	0.9600	0.9645	0.8904	0.204%
*n*_0_ = *n*_1_ = 100	0.9307	0.9406	0.8263	0.9552	0.9599	0.8395	0.065%
*n*_0_ = *n*_1_ = 500	0.9385	0.9480	0.6648	0.9534	0.9549	0.6657	0.068%
Overall	0.9303	0.9440	0.7841	0.9562	0.9600	0.7985	0.112%

**Table A2:** Summary of *p*_−_ confidence interval lengths where the cell values denote the confidence interval length for one fixed parameter and averaging over the remaining parameters. Hereby N-Approx and t-Approx are abbreviations for normal and t-approximation. *π* fix denotes the method of [1], where *π* is assumed to be fixed.

Fixed Parameter	N-Approx	Additive t-Approx	*π* fix	N-Approx	Logistic t-Approx	*π* fix
*π* = 0.05	0.1522	0.1554	0.1219	0.1533	0.1578	0.1231
*π* = 0.25	0.0798	0.0812	0.0595	0.0822	0.0844	0.0611
*π* = 0.5	0.0229	0.0243	0.0117	0.0254	0.0289	0.0122
*se* = 0.5	0.1036	0.1047	0.0632	0.1042	0.1065	0.0630
*se* = 0.75	0.0910	0.0920	0.0694	0.0920	0.0937	0.0696
*se* = 0.85	0.0781	0.0801	0.0655	0.0803	0.0832	0.0669
*se* = 0.9	0.0672	0.0710	0.0593	0.0712	0.0781	0.0624
*sp* = 0.5	0.1112	0.1137	0.0872	0.1131	0.1173	0.0882
*sp* = 0.75	0.0824	0.0844	0.0620	0.0844	0.0878	0.0632
*sp* = 0.85	0.0748	0.0766	0.0555	0.0768	0.0799	0.0567
*sp* = 0.9	0.0715	0.0732	0.0527	0.0734	0.0765	0.0539
*m_g_* = 100	0.1076	0.1101	0.0643	0.1108	0.1159	0.0655
*m_g_* = 500	0.0764	0.0781	0.0643	0.0778	0.0804	0.0655
*m_g_* = 1, 000	0.0710	0.0727	0.0643	0.0722	0.0748	0.0655
*n*_0_ = *n*_1_ = 50	0.1100	0.1140	0.0953	0.1136	0.1205	0.0979
*n*_0_ = *n*_1_ = 100	0.0862	0.0876	0.0675	0.0878	0.0899	0.0683
*n*_0_= *n*_1_ = 500	0.0587	0.0593	0.0302	0.0594	0.0607	0.0303
Overall	0.0850	0.0869	0.0643	0.0869	0.0904	0.0655 %
